# Medium cut-off dialyzer improves erythropoiesis stimulating agent resistance in a hepcidin-independent manner in maintenance hemodialysis patients: results from a randomized controlled trial

**DOI:** 10.1038/s41598-020-73124-x

**Published:** 2020-09-29

**Authors:** Jeong-Hoon Lim, Yena Jeon, Ju-Min Yook, Soon-Youn Choi, Hee-Yeon Jung, Ji-Young Choi, Sun-Hee Park, Chan-Duck Kim, Yong-Lim Kim, Jang-Hee Cho

**Affiliations:** 1 Division of Nephrology, Department of Internal Medicine, School of Medicine, Kyungpook National University, Daegu, South Korea; 2 Department of Statistics, Kyungpook National University, Daegu, South Korea

**Keywords:** Nephrology, Kidney diseases, Renal replacement therapy

## Abstract

The response to erythropoiesis stimulating agents (ESAs) is affected by inflammation linked to middle molecules in hemodialysis (HD) patients. We evaluated the effect of a medium cut-off (MCO) dialyzer on ESA resistance in maintenance HD patients. Forty-nine patients who underwent high-flux HD were randomly allocated to the MCO or high-flux group. The primary outcome was the changes of erythropoietin resistance index (ERI; U/kg/wk/g/dL) between baseline and 12 weeks. The MCO group showed significant decrease in the ESA dose, weight-adjusted ESA dose, and ERI compared to the high-flux group at 12 weeks (*p* < 0.05). The generalized estimating equation models revealed significant interactions between groups and time for the ESA dose, weight-adjusted ESA dose, and ERI (*p* < 0.05). Serum iron and transferrin saturation were higher in the MCO group at 12 weeks (*p* < 0.05). The MCO group showed a greater reduction in TNF-α and lower serum TNF-α level at 12 weeks compared to the high-flux group (*p* < 0.05), whereas no differences were found in the reduction ratio of hepcidin and serum levels of erythropoietin, erythroferrone, soluble transferrin receptor and hepcidin between groups. HD with MCO dialyzer improves ESA resistance over time compared to high-flux HD in maintenance HD patients. The MCO dialyzer provides superior removal of the inflammatory cytokine and thus improves iron metabolism in a hepcidin-independent manner.

## Introduction

Anemia is a frequent complication of end-stage renal disease (ESRD) and is associated with increased morbidity and mortality rates^1^. Anemia in ESRD has multifactorial causes, including erythropoietin deficiency, uremia-related inhibition of erythropoiesis, inflammation, and low dialysis adequacy^2,3^. Erythropoiesis stimulating agents (ESAs) and iron are used to treat anemia in ESRD patients. Anemia correction improves the left ventricular mass index and improves the quality of life^4–6^; however, the responses to ESA vary due to several reasons, such as iron deficiency, poor nutritional state, and chronic inflammation^3,7,8^.

Uremic toxins and associated chronic inflammation are known to affect iron metabolism in ESRD patients and interferes with the response to ESA^9–11^. There are uremic substances of various sizes that cause ESA resistance, such as hepcidin, indoxyl sulfate, asymmetric dimethylarginine, and inflammatory cytokines^12–15^. Conventional hemodialysis (HD) effectively removes small molecules, but has a limitation in removing middle and large molecules. The removal of conventional middle to large molecules is believed to improve ESA response; therefore, online hemodiafiltration (OL-HDF) was studied as a solution. Previous studies have reported that OL-HDF clears uremic toxins of middle molecular weight (MW), improving the ESA resistance^16–18^.

Newly introduced medium cut-off (MCO) dialyzers have uniformly distributed larger pores and a better capacity to remove middle molecules and inflammatory cytokines than high-flux dialyzers or even OL-HDF^19,20^. However, there is no clear evidence that demonstrates the effect of MCO dialyzers on ESA resistance in maintenance HD patients. This study aimed to evaluate whether HD with MCO dialyzer can improve the ESA resistance in chronic HD patients.

## Results

### Patient characteristics

All the enrolled patients completed the study except one patient who withdrew consent in the MCO group (Supplementary Figure S1). The baseline characteristics of the HD patients were compared in Table 1. The age, sex, residual renal function, type of dialyzer, dialysis method, comorbidities, and ESA treatment were well balanced between two groups.

**Table 1:** Baseline characteristics.

	MCO (n = 24)	High-flux (n = 25)	*p*
Age (years)	62.2 ± 13.7	63.8 ± 15.2	0.687
Sex, male n (%)	18 (75.0)	15 (60.0)	0.364
Body mass index (kg/m^2^)	22.0 ± 2.6	21.8 ± 3.8	0.812
Residual renal function^a^, n (%)	4 (16.7)	6 (24.0)	0.524
Dialysis vintage (months)	83.6 ± 49.7	70.8 ± 48.4	0.367
Dialysis frequency, n (%)			0.966
2 times per week	2 (8.3)	2 (8.0)	
3 times per week	22 (91.7)	23 (92.0)	
Dialyzer, n (%)			1.000
FX CorDiax 80	17 (70.8)	18 (72.0)	
FX CorDiax 60	7 (29.2)	7 (28.0)	
Blood flow rate (mL/min)	245.4 ± 20.8	235.2 ± 19.6	0.084
Dialysate flow rate (mL/min)	500	500	
Dialysis time (min)	238.4 ± 9.2	234.8 ± 12.3	0.259
spKt/V	1.6 ± 0.2	1.7 ± 0.2	0.296
Comorbid conditions, n (%)			
Hypertension	19 (79.2)	20 (80.0)	1.000
Diabetes	12 (50.0)	14 (56.0)	0.674
Pre-dialysis SBP (mmHg)	145.2 ± 17.7	145.5 ± 19.7	0.954
Pre-dialysis DBP (mmHg)	68.6 ± 17.1	66.0 ± 14.4	0.569
Post-dialysis SBP (mmHg)	136.9 ± 22.2	129.1 ± 24.0	0.245
Post-dialysis DBP (mmHg)	69.3 ± 12.4	61.8 ± 15.3	0.067
ESA dose (U/week)	8343.8 ± 5878.2	7110.0 ± 7160.4	0.514
Weight-adjusted ESA dose (U/kg/week)	133.9 ± 91.5	126.9 ± 125.8	0.826
ERI (U/kg/week/g/dL)	12.8 ± 8.7	12.6 ± 13.9	0.965

Data are shown as mean ± standard deviation or n (%). Difference was analyzed using Student’s *t*-test for continuous variables and Pearson’s chi-square test or Fisher’s exact test for categorical variables.

MCO, medium cut-off; SBP, systolic blood pressure; DBP, diastolic blood pressure; ESA, erythropoiesis stimulating agent; ERI, erythropoietin resistance index.

^a^Daily urine volume more than 100 mL.

### Comparison of the ESA resistance, biochemical and iron metabolism parameters

At baseline, the number of patients who used oral iron agents was similar between the MCO and high-flux groups; all of them maintained the oral iron agents during the study period. The number of patients who used parenteral iron did not differ between groups. There was no difference in the transfusion history and the types of used ESA between the MCO and high-flux groups (Table 2).

**Table 2:** Clinical information on the anemia management.

	MCO	High-flux	*p*
Number of patients using oral iron at baseline, n (%)	16 (66.7)	17 (68.0)	0.921
Number of patients using oral iron at 12 weeks, n (%)	16 (66.7)	17 (68.0)	0.921
Number of patients treated parenteral iron during study period, n (%)	3 (12.5)	8 (32.0)	0.102
Cumulative dose of parenteral iron per treated patients (mg)	600.0 ± 100.0	700.0 ± 297.6	0.426
Transfusion history during study period, n (%)	0	1 (4.0)	1.000
Used ESA type, n (%)			0.679
Epoetin alfa	13 (54.2)	17 (68.0)	
Darbepoetin alfa	10 (41.7)	7 (28.0)	
None	1 (4.2)	1 (4.0)	

Data are shown as mean ± standard deviation or n (%). Difference was analyzed using Student’s *t*-test for continuous variables and Pearson’s chi-square test or Fisher’s exact test for categorical variables.

MCO, medium cut-off; ESA, erythropoiesis stimulating agent.

**Figure 1:**
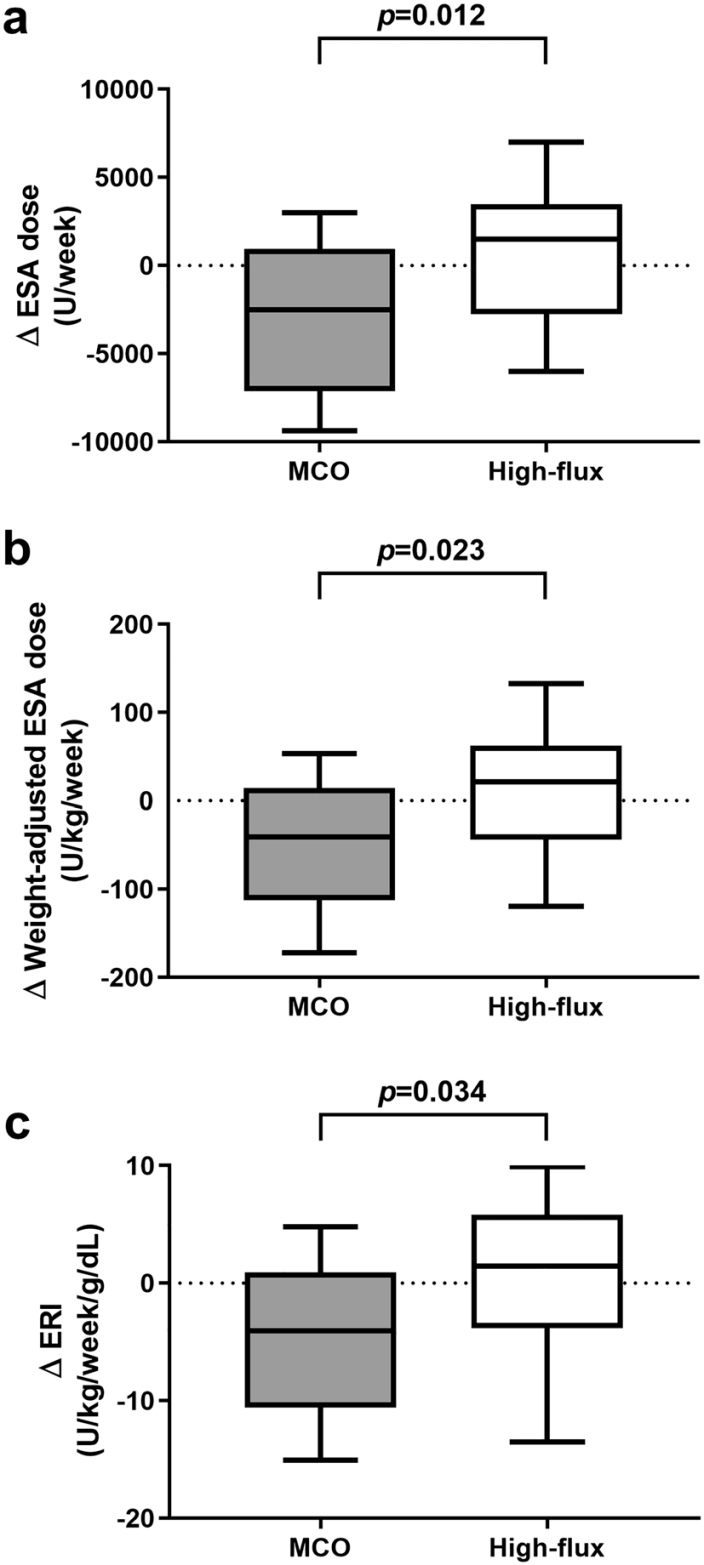
Comparison of the ESA, weight-adjusted ESA, and ERI difference. ESA, erythropoiesis stimulating agent; ERI, erythropoietin resistance index; MCO, medium cut-off.

Figure 1 displays the changes of ESA dose, weight-adjusted ESA dose, and erythropoietin resistance index (ERI) in groups. A comparison of differences in the baseline and 12 weeks values of ESA dose and weight-adjusted ESA dose showed significantly lower values in the MCO group than in the high-flux group (∆ ESA [U/wk]: − 3135.4 ± 4836.4 vs. 560.0 ± 5090.0, *p* = 0.012; ∆ weight-adjusted ESA [U/kg/wk]: − 49.8 ± 81.6 vs. 8.1 ± 90.2, *p* = 0.023). The difference (∆) of ERI was significantly lower in the MCO group than in the high-flux group (− 5.2 ± 7.8 vs. 0.1 ± 9.1 U/kg/wk/g/dL, *p* = 0.034).

**Figure 2:**
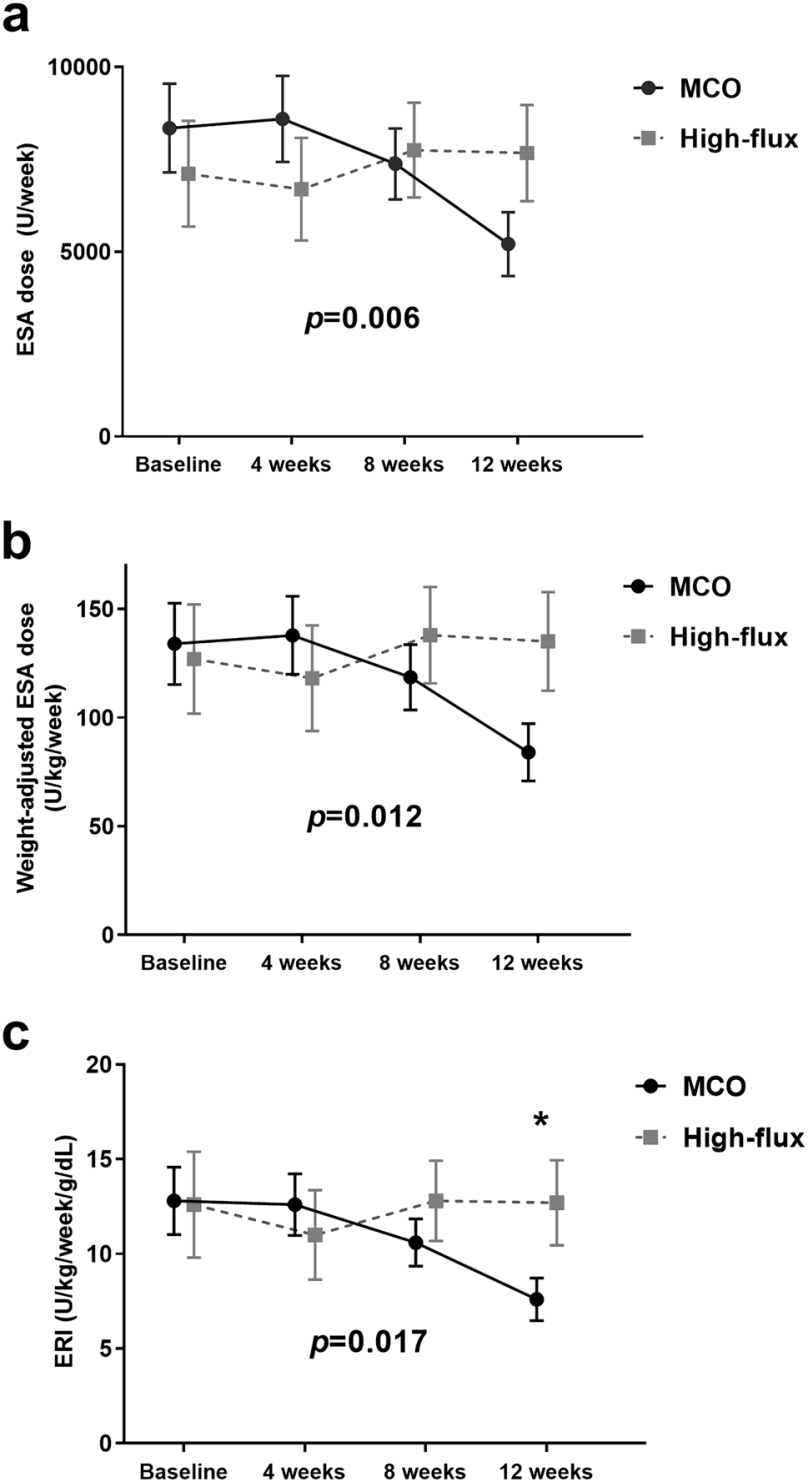
Serial changes in the ESA, weight-adjusted ESA, and ERI. The *p* values for the difference between groups from baseline to 12 weeks were calculated in the generalized estimating equation (GEE) models. Asterisk (*) indicates significant difference between groups at the time point (*p* < 0.05). ESA, erythropoiesis stimulating agent; ERI, erythropoietin resistance index; MCO, medium cut-off.

Figure 2 shows the monthly changes in the ESA dose, weight-adjusted ESA dose, and ERI levels. The generalized estimating equation (GEE) models revealed significant interactions between groups and time for the ESA dose, weight-adjusted ESA dose, and ERI levels from baseline to 12 weeks (*p* = 0.006, *p* = 0.012, and *p* = 0.017, respectively). The ERI at 12 weeks was significantly lower in the MCO group compared to the high-flux group (*p* = 0.048).

Biochemical data at baseline and at 12 weeks after randomization, and changes during study period are shown in Table 3. At the start and end of the study, serum hemoglobin, albumin, and high-sensitivity C-reactive protein levels were similar between the groups, and the changes during the study were not significant. Iron parameters were not different at baseline; however, the serum iron and transferrin saturation (TSAT) levels were higher in the MCO group at 12 weeks (iron [μg/dL]: 72.1 ± 25.4 vs. 55.9 ± 25.0, *p* = 0.029; TSAT [%]: 34.0 ± 15.0 vs. 25.3 ± 11.9, *p* = 0.031). Other parameters of iron metabolism, such as, erythroferrone, erythropoietin, and soluble transferrin receptor (sTfR), were not different at baseline and 12 weeks after randomization. Serum hepcidin level also showed no difference between groups at baseline and at 12 weeks. Tumor necrosis factor-alpha (TNF-α) level did not differ at baseline; after 12 weeks, the level was significantly lower in the MCO group than in the high-flux group (16.3 ± 3.4 vs. 19.0 ± 4.8 pg/mL, *p* = 0.027). However, the changes of these biochemical and iron metabolism parameters did not show significant difference between groups (all *p* > 0.05).

**Table 3:** Comparisons of the biochemical and iron metabolism parameters.

	Baseline			12 weeks			Difference (Δ) between baseline and 12 weeks		*p* for difference (Δ) between groups
	MCO	High-flux	*p*	MCO	High-flux	*p*	MCO	High-flux	
Hemoglobin (g/dL)	10.6 ± 0.9	10.7 ± 1.1	0.859	10.9 ± 0.9	11.0 ± 1.0	0.697	0.2 (− 0.5, 0.0)	0.0 (− 0.3, 0.9)	0.841
Albumin (g/dL)	4.11 ± 0.38	4.06 ± 0.33	0.635	3.98 ± 0.27	4.04 ± 0.33	0.450	− 0.05 (− 0.30, 0.00)	− 0.10 (− 0.20, 0.15)	0.252
hs-CRP (mg/dL)	0.11 (0.03, 0.26)	0.18 (0.05, 0.71)	0.704	0.13 (0.04, 0.46)	0.22 (0.06, 1.30)	0.250	0.00 (-0.10, 0.17)	0.06 (− 0.03, 0.78)	0.161
Ferritin (ng/mL)	161.1 (70.1, 305.3)	90.3 (38.6, 205.9)	0.156	123.9 (57.9, 312.2)	158.1 (59.5, 284.2)	0.904	19.2 ± 173.6	19.1 ± 151.7	0.998
Iron (μg/dL)	66.1 ± 25.0	59.6 ± 29.8	0.410	72.1 ± 25.4	55.9 ± 25.0	**0.029**	2.49 (− 5.9, 15.8)	− 1.6 (− 15.8, 4.0)	0.131
TIBC (μg/dL)	221.4 ± 37.8	234.8 ± 51.7	0.309	221.1 ± 46.3	227.1 ± 33.9	0.607	− 0.3 ± 36.3	− 7.7 ± 32.2	0.455
TSAT (%)	30.6 ± 12.3	26.1 ± 11.9	0.196	34.0 ± 15.0	25.3 ± 11.9	**0.031**	− 0.6 (− 4.4, 8.1)	0.8 (− 8.5, 3.7)	0.325
ERFE (pg/mL)	402.5 ± 122.3	360.3 ± 136.0	0.259	438.3 ± 123.0	386.0 ± 116.2	0.133	53.0 (− 38.3, 95.8)	44.6 (0.8, 73.6)	0.660
EPO (mU/mL)	9.5 (6.7, 16.0)	11.9 (5.0, 18.5)	0.818	10.1 (4.6, 19.9)	7.9 (5.3, 15.3)	0.741	− 2.3 (− 6.5, 6.2)	0.5 (− 9.5, 3.2)	0.889
sTfR (nmol/L)	16.7 (12.8, 23.5)	17.8 (13.6, 23.7)	0.617	16.4 (11.3, 21.9)	18.5 (11.1, 23.6)	0.660	− 1.4 ± 8.3	− 1.5 ± 10.1	0.981
Hepcidin (ng/mL)	46.8 ± 36.9	32.4 ± 27.3	0.128	42.1 ± 23.8	44.9 ± 26.3	0.688	− 3.5 ± 31.1	12.3 ± 27.0	0.063
TNF-α (pg/mL)	17.9 ± 5.0	18.0 ± 4.7	0.915	16.3 ± 3.4	19.0 ± 4.8	**0.027**	− 1.6 ± 4.3	1.0 ± 5.7	0.079

Data are shown as mean ± standard deviation or median (interquartile range). Difference was analyzed using Student’s *t*-test for normally distributed variables and Mann-Whiney *U* test for non-normally distributed variables. Values in bold indicate statistically significant results.

MCO, medium cut-off; hs-CRP, high-sensitivity C-reactive protein; TIBC, total iron binding capacity; TSAT, transferrin saturation; ERFE, erythroferrone; EPO, erythropoietin; sTfR, soluble transferrin receptor; TNF-α, tumor necrosis factor-alpha.

Table 4 shows the results of the multivariate linear regression analysis for the ERI difference (∆). HD with MCO dialyzer, change of high-sensitivity C-reactive protein (hs-CRP), and parenteral iron use were independently associated with ERI difference (MCO dialyzer: β =  − 0.38, *p* = 0.009; change of hs-CRP: β = 0.29, *p* = 0.034; parenteral iron use: β =  − 0.40, *p* = 0.006).

**Table 4:** Multivariate linear regression analysis of the factors associated with differences in the ERI.

Variables	Β	SE	*β*	*p*
Dialyzer type	− 6.55	2.38	− 0.38	**0.009**
Age	− 0.08	0.08	− 0.13	0.340
Sex	− 1.27	2.56	− 0.07	0.624
Dialysis vintage	− 0.04	0.03	− 0.22	0.127
Change (Δ) of hs-CRP	2.67	1.22	0.29	**0.034**
Use of parenteral iron agents	− 8.41	2.91	− 0.40	**0.006**

The reference dialyzer is high-flux dialyzer and sex is male. Values in bold indicate statistically significant results.

ERI, erythropoietin resistance index; hs-CRP, high-sensitivity C-reactive protein.

### Comparison of the reduction ratio of serum hepcidin and TNF-α

**Figure 3:**
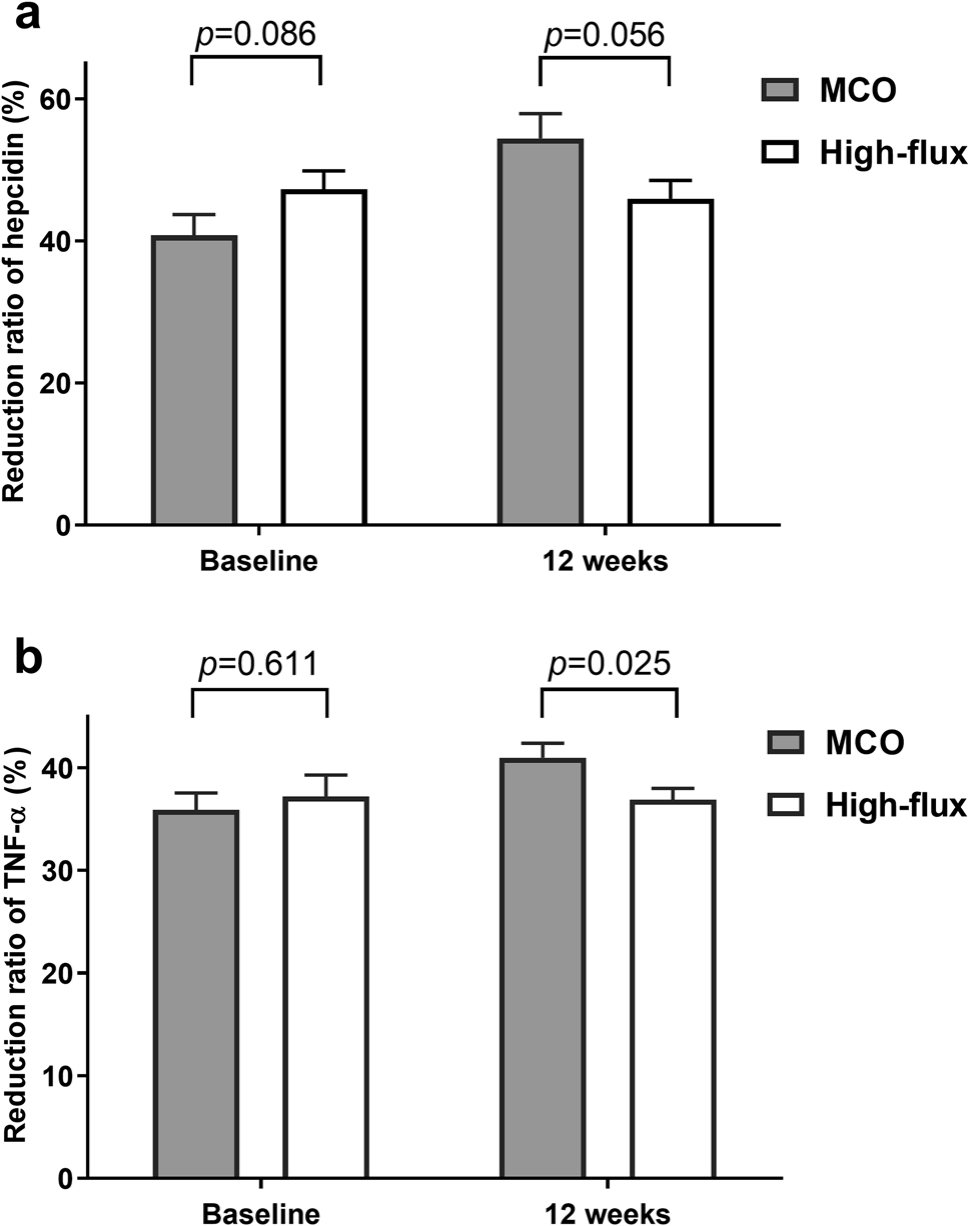
Reduction ratio of serum hepcidin and TNF-α at baseline and at 12 weeks. TNF-α, tumor necrosis factor-alpha; MCO, medium cut-off.

The reduction ratio (RR) of serum hepcidin was similar in the two groups at baseline; the RR at 12 weeks was also not different in the MCO group compared to that in the high-flux group (Fig. 3a). RR of TNF-α in the groups was similar at baseline; however, after 12 weeks, it was higher in the MCO group than in the high-flux group (41.0 ± 6.8% vs. 36.9 ± 5.4%, *p* = 0.025; Fig. 3b).

## Discussion

The present study showed that HD with MCO dialyzer reduced ESA resistance compared to high-flux HD. MCO group showed a greater decrease in the ERI than the high-flux group. The serum iron level and TSAT at 12 weeks was higher in the MCO group despite the comparable use of parenteral iron. The MCO dialyzer had advantages of better removal of TNF-α. This suggests that HD using MCO dialyzer causes reduced inflammation that may improve the iron metabolism and ESA response.

In the present study, changing from high-flux dialyzer to MCO dialyzer improved the ESA resistance as compared to HD with high-flux dialyzer. Several reports have emphasized the importance of ESA resistance in patients with HD, not only because of economic reasons, but also because ESA hyporesponsive HD patients have shown increased all-cause mortality and cardiovascular complications^1,21–24^. According to our results, HD with MCO dialyzer significantly decreased the amount of ESA during the study period as compared to the high-flux HD. Although we did not evaluate the impact of improved ESA resistance on mortality and cardiovascular events, it might have a positive effect on the prognosis in the long-term because a previous study has demonstrated an association between ESA responsiveness and mortality in ESRD patients^25^.

The improvement in ESA resistance using MCO dialyzer may be attributable to the improved removal of inflammatory cytokine. There is a consensus on the connection between ESRD and inflammation^9^. Under uremic conditions, chronic inflammation may induce an enhanced state of T-cell activation that leads to ESA resistance^18,26^. In addition, bone marrow erythropoiesis suppression by the inflammatory cytokines is suggested as a cause of anemia in HD patients^27^. TNF-α is a representative pro-inflammatory cytokine that has a MW of 17.3 kDa and is usually elevated in patients with chronic kidney disease^28–31^. TNF-α causes anemia by inhibiting erythroid-precursor proliferation and promoting hypoferremia^32,33^. We confirmed that MCO dialyzer is not only better for TNF-α removal, but also for lowering the TNF-α level at 12 weeks of treatment as compared to high-flux HD. Zickler et al. also reported that the MCO dialyzer lowered the TNF-α and IL-6 mRNA expression in peripheral blood to a greater extent than a high-flux dialyzer^20^. They showed that even after only 4 weeks of HD with MCO dialyzer, chronic inflammation markers were significantly reduced. However, they did not evaluate the RR of TNF-α and could not prove the difference of TNF-α between MCO and high-flux groups. Our study had a longer study period and demonstrated that MCO dialyzer was more effective for removing serum TNF-α than a high-flux dialyzer. In contrast to our findings, a recent French cross-over study^34^, showed no difference of ERI between HD with MCO dialyzer and high-flux dialyzer after 12 weeks of dialysis. The cross-over study design has a limitation to evaluate the ERI changes, because the legacy effect of the dialyzer has confounded the effects on the ESA resistance. Therefore, our research is the first study to demonstrate the ERI improvement without confounding variables.

To clearly identify the association between TNF-α and ESA resistance, we evaluated the changes in erythropoiesis and iron metabolism-related parameters, such as hepcidin, erythroferrone, erythropoietin, and sTfR. TNF-α results in hypoferremia through both hepcidin-independent and -dependent mechanisms. In the hepcidin-independent pathway, TNF-α relocalizes ferroportin in small bowel enterocytes, thus it causes systemic iron deficiency^33,35,36^. Therefore, reduced TNF-α level following increased removal of TNF-α by the MCO dialyzer might improve utilization of iron in HD patients. However, because of an intracellular and vesicular distribution of ferroportin, it is difficult to accurately measure ferroportin activity^37^. Further studies are needed to clarify the pathophysiology between reduced TNF-α and changes in ferroportin expression.

**Figure 4:**
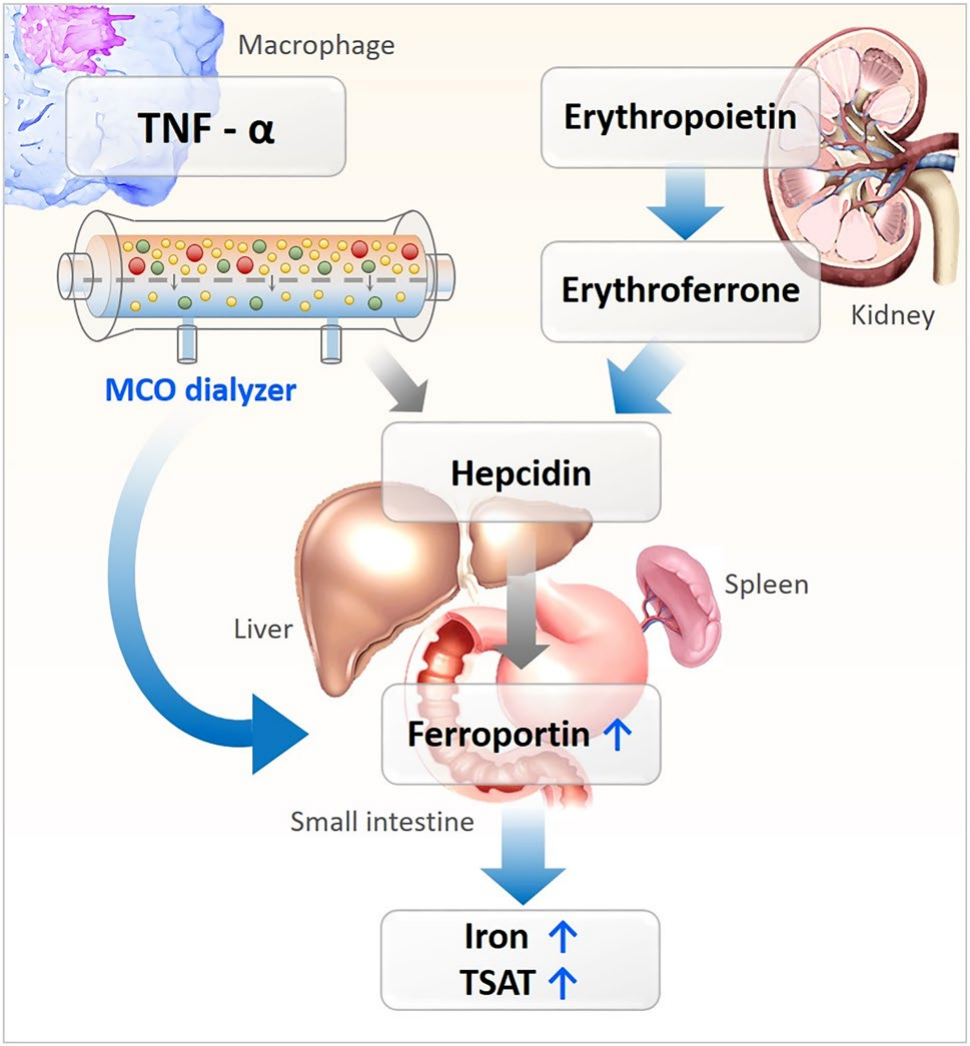
The iron metabolism regulatory pathway. Blue arrows indicate dominant effects. TNF-α, tumor necrosis factor-alpha; MCO, medium cut-off; TSAT, transferrin saturation.

Hepcidin is a small-sized middle molecular uremic toxin that is related to ESA resistance^38^. The serum hepcidin level is controlled by both inflammation and erythropoietin^39^. The RR and the level of hepcidin did not differ between the two groups because of hepcidin’s small MW. In addition, the upper regulators of hepcidin, erythroferrone and erythropoietin, also remained unchanged after switching to the MCO dialyzer, and this might be related to their large molecular weights that are not removed by the HD with MCO dialyzer. We speculated that the increased removal of TNF-α did not affect the hepcidin level directly because TNF-α is not a dominant regulator of hepcidin as a previous study has identified^40^ or the decrease of TNF-α was not sufficient to affect hepcidin level. Moreover, we maintained iron homeostasis via proper ESA use and iron supplement as per the guideline. Thus, the iron status did not exert much influence on hepcidin level. Figure 4 depicts our hypothesis that HD with MCO dialyzer increases the removal of inflammatory cytokines, such as TNF-α, and it improves the iron status by both hepcidin-independent and dependent pathway, although independent pathway was more dominant in our study. As a result, ESA responsiveness would have improved in MCO HD patients.

The improvement of ESA resistance through the removal of middle molecules in HD patients has been of interest to researchers. Several studies have evaluated the effect of dialysis membranes or dialysis methods on ESA resistance and have reported conflicting results. OL-HDF is superior in terms of the removal of middle to large molecules than conventional dialysis^41–43^. Some randomized controlled trials and cross-over studies have confirmed that OL-HDF reduced ESA resistance more effectively than conventional HD^16–18^. They explained that the increased removal of middle molecules by convection would have improved the uremic status and increased the ESA response. Similarly, in our study, MCO dialyzer showed greater removal of middle-sized inflammatory cytokine and improved the ESA resistance. In addition, MCO dialyzer has also been verified as an independent predictor that reduces ERI. HD using MCO dialyzer does not involve a higher cost, specific hardware, or unusual nursing skills as OL-HDF^44,45^; therefore, it will be useful for HD patients with ESA resistance in dialysis centers that cannot perform OL-HDF.

The strength of this study is that this is the first study based on the randomized controlled trial that has evaluated the effect of HD using MCO dialyzer on ESA resistance. However, there are certain limitations of this study. First, the number of registered patients was small, and the study duration was not long enough to get definite results. Second, although anemia-related parameters, such as iron, TSAT, and TNF-α, were significantly different at 12-week, the within-group differences were not significant for these parameters. Therefore, we could not conclude that the changes of these parameters were significant after the intervention. These inconsistent results of anemia-related parameters by different statistical tests may be due to the small number of patients. To clearly identify the effect of MCO dialyzer on anemia-related parameters and prove our hypothesis on ESA resistance, follow-up studies with a larger number of patients are required. Third, this study was not blinded to clinicians and could have affected the prescription of ESA and iron supplementation. Fourth, the detailed mechanism regarding how ESA response was improved by increased removal of middle molecules remains unclear. Nevertheless, we believe that our study will be the basis for a future well-designed, long-term, large-scale study.

In conclusion, the MCO dialyzer achieved more improvement in ESA resistance than the high-flux dialyzer. The MCO dialyzer removed more quantity of the inflammatory cytokine such as TNF-α than the high-flux dialyzer, potentially influencing the iron metabolism.

## Methods

### Patients and study design

This is a post-hoc analysis of the prospective, randomized, controlled, open-label trial to compare the ESA resistance between MCO and high-flux HD patients. The original randomized controlled trial was designed to evaluate the effectiveness of MCO dialyzer on quality of life compared to high-flux dialyzer^46^. Chronic maintenance HD patients treated with high-flux dialyzer at the Kyungpook National University Hospital were enrolled from July 2018 and followed up for 12 weeks; the study ended in January 2019. The trial was registered with the Clinical Research Information Service (CRiS) at the Korea Centers for Disease Control and Prevention (KCT0003026; registration date: 25/07/2018), and detailed information regarding the inclusion and exclusion criteria are provided in our previous study^46^ and CRiS website (https://cris.nih.go.kr).

We randomly allocated the patients in a 1:1 ratio to the MCO dialyzer and high-flux dialyzer groups. Randomization was conducted as per the random number table that was provided by a blinded statistician. The study patients and clinicians were immediately allocated the respective groups as per the randomization. The MCO group changed their dialysis membrane from a high-flux dialyzer (FX CorDiax 80 or 60; Fresenius Medical Care Deutschland, Bad Homburg, Germany) to a MCO dialyzer (Theranova 400; Baxter International Inc., Hechingen, Germany). The control group continued their treatments with the high-flux dialyzer. There was no change in the dialysis prescription for dialysis time per session, dialysis frequency per week, blood flow rate, and dialysate flow rate during the study period.

The target serum hemoglobin level was 11 g/dL, and the same nephrology physician prescribed the ESA and iron agents as per the KDOQI and KDIGO guidelines^47,48^. The ESA and parenteral iron were administered via the venous line at the end of the HD session. Oral iron agents were prescribed if necessary to maintain ferritin level > 100 ng/mL and TSAT > 20%. If the patient’s ferritin level or TSAT failed to reach the target hemoglobin level after being administered oral iron agents, parenteral iron sucrose was given.

The study was conducted as per the ethical principles of the Declaration of Helsinki; the Institutional Review Board of the Kyungpook National University Hospital approved the study protocol (KNUH 2017–11-024). Written informed consent was obtained from all the patients before inclusion.

### Data collection and analyses

#### Clinical data

Baseline demographics, comorbid diseases, biochemical data, and dialysis information were collected at the time of enrollment. Comorbid hypertension was defined by the European Society of Cardiology and the European Society of Hypertension (ESC/ESH) as blood pressure ≥ 140/90 mmHg^49^; comorbid diabetes was defined by use of glucose-lowering medication or glycated hemoglobin ≥ 6.5% or random plasma glucose ≥ 200 mg/dL^50^. Dialysis information and biochemical data were reassessed at 12 weeks after randomization. The use of iron agents and transfusion data during the study period were recorded. The ESA doses during the 12-week study period were recorded.

#### ESA resistance measurement

To evaluate the ESA resistance, we utilized the ERI that was calculated as the mean weekly weight-adjusted ESA dose divided by the hemoglobin level^23,51^; the level was measured every 4 weeks. Patients used epoetin alfa or darbepoetin alfa as ESA, and the ESA types remained unchanged during the study period. The dose conversion ratio of darbepoetin alfa to epoetin alfa was 1:200^18^.

#### Sampling and analyses

Blood samples for the measurement of biochemical markers were obtained at the start of a midweek dialysis session. All the baseline samples were collected under high-flux HD. The samples were collected in individual serum tubes and separated via centrifugation. Then, the serum samples were immediately frozen and stored at − 80 °C until use. Concentrations of the iron metabolism and inflammatory molecules were measured using commercially available ELISA kits: erythroferrone (MW 37.3 kDa) with the Human protein FAM132B ELISA Kit (MyBioSource, San Diego, CA, USA), erythropoietin (MW 30.4 kDa) with the Quantikine IVD Human Erythropoietin ELISA Kit (R&D systems, Minneapolis, MN, USA), sTfR (MW 84.9 kDa) with the Quantikine IVD Human sTfR ELISA KIT (R&D systems, Minneapolis, MN, USA), hepcidin (MW 2.8 kDa) with the Hepcidin 25 bioactive HS ELISA Kit (DRG Instruments, Marburg, Germany), and TNF-α (MW 17.3 kDa) with Human TNF-α ELISA Kit (R&D systems, Minneapolis, MN, USA). All the assays were performed as per the manufacturer’s protocols. To measure the RR, post-dialysis samples were collected using the slow-flow method^52^.

#### Reduction ratio calculation

RR of serum hepcidin and TNF-α was calculated using pre-to-post-dialysis serum concentration, and the Bergstrom and Wehle formula^53^ was used to compensate the hemoconcentration during HD. The detailed calculation method has been shown in our previous study^46^.

#### Study outcomes

The primary outcome was the changes of ERI between baseline and 12 weeks of treatment that reflects ESA resistance change. The secondary outcomes were iron- and anemia-related markers, and reduction ratios of the iron regulator (hepcidin) and the inflammatory cytokine (TNF-α) at baseline and 12 weeks of treatment.

### Statistical analyses

The patients who completed the study were included in the analysis. The Kolmogorov–Smirnov test was applied to analyze the normal distribution of variables. All the variables were reported as means ± standard deviation values, median (interquartile range) values, or number (percentage, %), based on the nature and distribution of the variables. Student’s *t* tests and Mann–Whitney *U* tests were applied to determine the differences between the continuous variables as appropriate; for categorical variables, Pearson’s chi-square tests or Fisher’s exact tests were utilized. For repeated measured data (ESA dose, weight-adjusted ESA dose, and ERI), GEE was used to assess the effect of MCO dialyzer for ESA resistance over 12 weeks. Differences in the RR between baseline and 12 weeks in each group were compared using paired *t* tests. Multiple linear regression analysis was used to identify the factors associated with ERI changes (∆). Statistical analyses were performed with SPSS version 22.0 (SPSS, Chicago, IL, USA). A *p* value < 0.05 was considered to indicate statistical significance.

## Supplementary information

Supplementary Figure.

## Data Availability

The datasets generated during and/or analysed during the current study are available from the corresponding author on reasonable request.
