# Supplementary material for: Medium cut-off dialyzer improves erythropoiesis stimulating agent resistance in a hepcidin-independent manner in maintenance hemodialysis patients: results from a randomized controlled trial
Source: Sci Rep. 2020 Sep 29;10:16062. doi: 10.1038/s41598-020-73124-x (PMC7524751; doi:10.1038/s41598-020-73124-x)

Medium cut-off dialyzer improves erythropoiesis stimulating agent resistance in a hepcidin-independent manner in maintenance hemodialysis patients: results from a randomized controlled trial

Jeong-Hoon Lim[1], Yena Jeon[2], Ju-Min Yook[1], Soon-Youn Choi[1], Hee-Yeon Jung[1], Ji-Young Choi[1], Sun-Hee Park[1], Chan-Duck Kim[1], Yong-Lim Kim[1], and Jang-Hee Cho[1,*]

[1]Department of Internal Medicine, School of Medicine, Kyungpook National University, Daegu, South Korea

[2]Department of Statistics, Kyungpook National University, Daegu, South Korea

Supplementary Information Listing:

Supplementary Figure S1

[*]Corresponding author: Jang-Hee Cho, MD, PhD

Associate Professor

Division of Nephrology, Department of Internal Medicine

Kyungpook National University Hospital, 130 Dongdeok-ro, Jung-gu, Daegu, 41944, Korea

Tel: +82-53-200-5550

Fax: +82-53-426-2046

Email: jh-cho@knu.ac.kr

Supplementary Figure S1. CONSORT flow chart of patient inclusion.

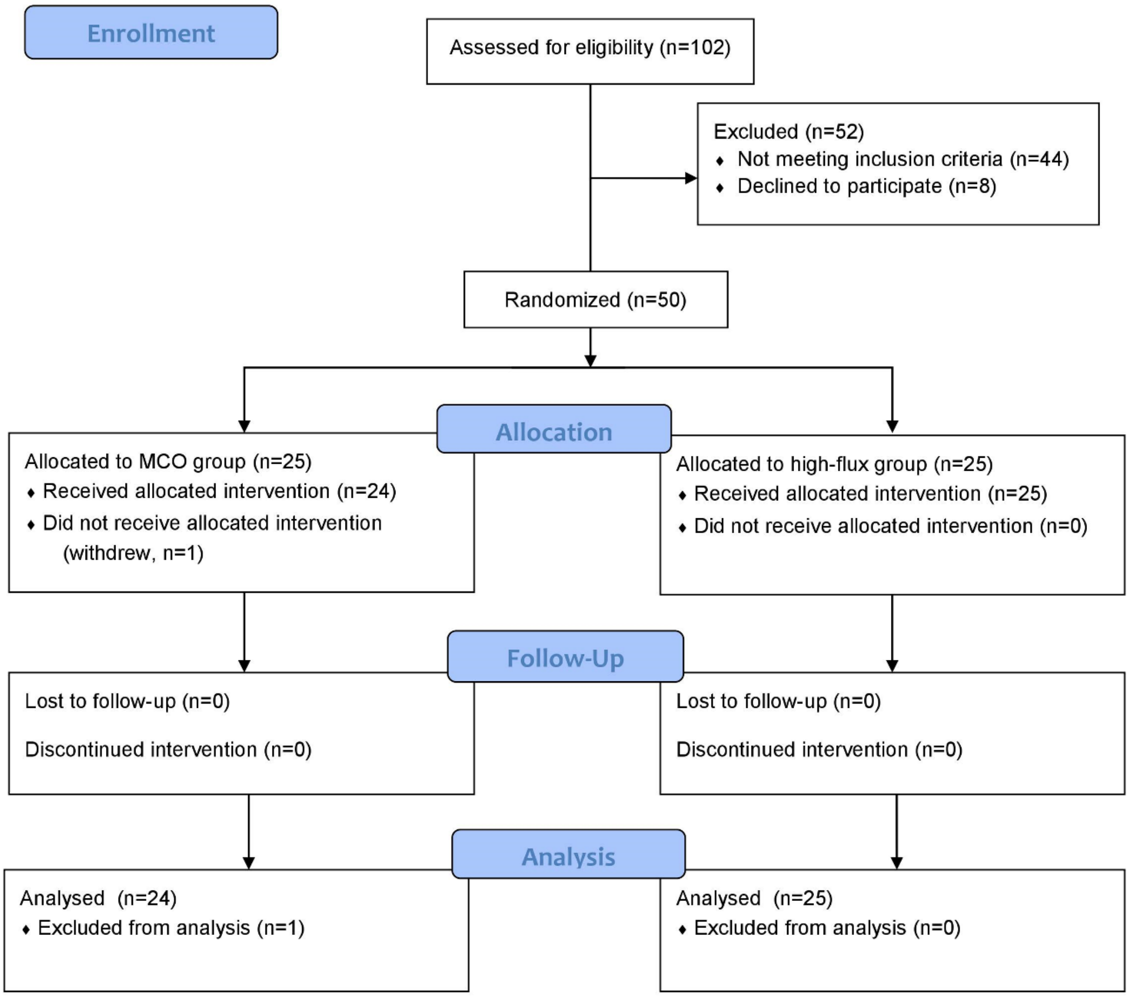

Supplement: Supplementary file 1 — Supplementary Figure. [file 41598_2020_73124_MOESM1_ESM.pdf]
